# Investigating the mental health of university students during the COVID-19 pandemic in a UK university: a machine learning approach using feature permutation importance

**DOI:** 10.1186/s40708-023-00205-8

**Published:** 2023-10-10

**Authors:** Tianhua Chen

**Affiliations:** School of Computing and Engineering, University of Huddersfield, Huddersfield, West Yorkshire HD1 3DH UK

**Keywords:** Mental Wellbeing, Mental Health, Student Wellbeing, Artificial Intelligence, Machine Learning

## Abstract

Mental wellbeing of university students is a growing concern that has been worsening during the COVID-19 pandemic. Numerous studies have gathered empirical data to explore the mental health impact of the pandemic on university students and investigate factors associated with higher levels of distress. While the online questionnaire survey has been a prevalent means to collect data, regression analysis has been observed a dominating approach to interpret and understand the impact of independent factors on a mental wellbeing state of interest. Drawbacks such as sensitivity to outliers, ineffectiveness in case of multiple predictors highly correlated may limit the use of regression in complex scenarios. These observations motivate the underlying research to propose alternative computational methods to investigate the questionnaire data. Inspired by recent machine learning advances, this research aims to construct a framework through feature permutation importance to empower the application of a variety of machine learning algorithms that originate from different computational frameworks and learning theories, including algorithms that cannot directly provide exact numerical contributions of individual factors. This would enable to explore quantitative impact of predictors in influencing student mental wellbeing from multiple perspectives as a result of using different algorithms, thus complementing the single view due to the dominant use of regression. Applying the proposed approach over an online survey in a UK university, the analysis suggests the past medical record and wellbeing history and the experience of adversity contribute significantly to mental wellbeing states; and the frequent communication with families and friends to keep good relationship as well as regular exercise are generally contributing to improved mental wellbeing.

## Introduction

Mental wellbeing of university students has been a growing concern that the UK House of Commons Library Briefing of December 2020 recently summarised that there has been sixfold increase in student mental ill health since [1]. The report has further concluded that COVID-19 pandemic has had a negative impact on student’s mental health, as confirmed in many studies [2–4], with majority of students reporting that their mental health and wellbeing has deteriorated and they have higher levels of anxiety and lower levels of happiness than the general population [1]. This is mainly due to numerous specific challenges presented by the pandemic including the forced conversion of more online learning that students found challenging to engage [5]; the distancing measures that limited opportunities for socialising and establishing relationships [6], and increased uncertainties on job market and career prospect [7]. In response to the unprecedented situation, psychologists and researchers are increasingly raising concerns and gathering empirical data to explore and understand the mental health impact of the pandemic on university students and investigate factors associated with higher levels of distress.

A cross-national research [8] shows that the student population were experiencing mental wellbeing issues that had to ask for regular psychological support. Another online survey conducted among university students in Bangladeshi[9] shows that students experienced high levels of anxiety and depression with low mental health statuses, while transitioning to the new norms of the pandemic in April 2020. In an online survey study [10], it has been found that social media has been frequently used as a coping mechanism, which was associated with greater negative influences on academic performance and stress levels for female undergrads; whist males students experienced greater negative impacts through using cannabis. Another online survey for a UK university [11] also identified high levels of anxiety and depression among university students, with over half surveyed experiencing levels above the clinical cut offs, particularly female students who were scoring significantly higher than males. In a large online study [12] conducted among Greek-speaking university students, it is identified that there were significant direct impact of the pandemic on participants’ financial status on satisfaction with life and indirect influence on participants’ financial status and academic performance, respectively, on satisfaction with life through general mental health. Longitudinal studies comparing mental health before and during the pandemic are also prevalent. For example, a study at one UK university [13] intended to explore the mental wellbeing landscape of undergrads between the first national lockdown and before the pandemic. They found that more than a third of participants could be classified as clinically depressed at lockdown, a significant increase from 15% before the pandemic, which was highly correlated with worse sleep quality. Another study [14] demonstrated through filling online survey at two time points that students with preexisting mental health concerns showed improving or similar mental health during the pandemic compared to one year before. In contrast, students without preexisting mental health issues were more likely to show worse mental wellbeing, which coincided with increased social isolation among theses students. A more recent study [15] explored determinants and predictors of mental health and concluded that during lockdown, students from low-income families experienced higher anxiety than high-income groups; inactive students were less likely to be anxious than active students, and female students were more likely to be depressed compared to male students. After the lockdown, students from low-income families had more odds of being anxious and depressed than the high-income families.

Among the conducted research, it has been observed that the means of circulating online questionnaire surveys among groups of target students remains a dominating approach to obtain the research data, be it a one-off deployment [9, 11, 13, 14] or longitudinal studies across several milestones [13, 14, 16]. The choices of questionnaire surveys are typically consisted of questions in relation to individual students such as demographics, personal habits and patterns in the context of COVID-19. With respect to mental wellbeing states of research interest, the employment of existing psychological measures remains a prevalent approach to study students’ mental health, e.g., the PHQ9 for depression [17], GAD7 for anxiety [18], BRS6 for resilience [19]. This is usually followed by results analysis, whereby the regression analysis has been extensively utilised to evaluate the predictive capabilities of the independent variables with respect to a dependent variable of interest. While regression has been a prevalent choice for its straightforward readability to interpret and understand the impact of independent factors on a variable of interest, the generated models could be significantly influenced by outliers [20], e.g., some students can be fatigued and careless occasionally when facing dozens of questions in a questionnaire. The regression analysis may further be limited in cases where two or more variables are highly correlated [20], hence asking for a prior check on variable correlation that usually does not take place when designing a questionnaire. Moreover, the particular choice of linear regression also remains a prevalent choice for regression analysis in practice [11, 13, 21], with its linear assumption clearly limiting the model to work with complex scenarios where data are non-linearly distributed. These observations motivate the underlying research to work on alternative computational methods to investigate the questionnaire data.

Machine learning [22], with many successful applications in numerous domains including the health and mental wellbeing area [23, 24], is a recent computational paradigm that aims to automatically learn patterns hidden in the data. Supervised learning, as one of the broad learning categories, aims to build a computational model that can best map the given set of inputs to the desired outputs for predictive analysis. Apart from regression as a traditional statistical method, there exists various alternative frameworks in learning these data-driven models. For instance, the construction of decision tree algorithm is based on information theory [25]; the support vector machine (SVM) is one of the most robust prediction methods based on Vapnik–Chervonenkis computational learning theory [26]; the K-nearest neighbours algorithm is an instance-based learning approach that predicts the output directly utilising a set of nearest neighbours [27]; the neural networks are based on a collection of connected artificial neurons, loosely modelling the neurons in a biological brain [28]; the ensemble learning framework [29] adopts multiple algorithms to obtain better predictive performance than from any of the constituent learning algorithms alone, with successful algorithms including the random forest [30] and gradient boosted trees [31] that are ensembles of decision trees based on bagging and boosting strategy, respectively. The rich variety of machine learning approaches that originate from different learning frameworks and theories could potentially provide views different from and complement that as a result of the dominant use of regression analysis. This motivates the underlying research to apply influential machine learning algorithms to explore impact of different predictors in influencing student mental wellbeing.

In working towards analysing questionnaire surveys for student mental wellbeing, it is desirable to output predictors that are compelling as well as their exact numerical contributions so that these insights can be further exploited to inform decision-making and policy generation. In light of it, it is natural to consider machine learning algorithms that could directly assess feature significance. The popular choices include regression and in machine learning, predominately, a set of tree based approaches such as decision tree, random forest and gradient boosted tree. However, one major limitation of directly employing machine learning algorithms lies in the limited number of choices that could directly compute feature significance, which is in contrast with the fundamental motivation of this research, i.e. to assess feature significance from a set of learning algorithms as diverse as possible. Another limitation of the direct application of a certain algorithm lies in the its inherent drawbacks. For instance, the decision tree is known unstable that a small change in the data can lead to a completely different tree generated, hence the feature significance can be largely affected by noisy data, which could also cause the model to malfunction. Significance provided by random forest may be misled by high carnality features, i.e. features with many unique values.

In order to gain access to as many diverse learning algorithms as possible while overcoming potential drawbacks embedded inherently with certain learning algorithms, this research aims to adopt the strategy of feature permutation importance. The idea, which was first proposed in the construction of random forest algorithm [30], randomly shuffles a single variable of the data, leaving the target and all other predictors in place. The numerical significance of a feature can then be defined as the decrease in a model score when a single feature value is randomly shuffled [30]. This is due to randomly re-ordering a single predictor should cause less accurate predictions, since the resulting data no longer correspond to anything observed in the real world. Model accuracy especially suffers if a variable is shuffled that the computational model relies on heavily for predictions. For instance, the depression level of a student may more depend on the whether they have a good family relationship than the ethical background; hence the distortion of relationship values could potentially cause terrible model predictions; whereas the random change of ethical background may not make the model suffer as much. Being able to break the relationship between the feature and the target, the drop in the model score is therefore indicative of how much the model depends on the feature. These observations motivate the underlying research to adopt the permutation strategy to compute the feature importance. Furthermore, the permutation importance being model agnostic, is a post-mortem approach that works after the algorithm has fit the data; hence, this strategy empowers the access to a range of machine learning algorithms, including those that cannot directly compute numerical variable importance.

## Materials

The materials used to demonstrate the proposal of feature permutation importance with machine learning technique comes from an on-going research project on understanding the impact of pandemic and lockdown on university student mental wellbeing. Published in early 2022 [11], the initial research was meant to investigate the mental wellbeing of higher education students at an early stage in the COVID-19 pandemic and to investigate factors associated with higher levels of distress. A cross-sectional online questionnaire survey was deployed at a university with almost 20,000 students in the North of England, UK. A total of 1173 valid responses from both undergraduates and postgraduates across all seven schools at the university were collected without any missing values (the survey required students to fill in all questions before they can submit). The data were collected in the period between 26.06.2020 and 30.07.2020, soon after the measures of first national lockdown in England starting from 23.02.20 and eased from 01.06.20. Following the relevant guidelines and regulations of University of Huddersfield, the research was performed following the approval by the ethical committee panel of School of Computing and Engineering, University of Huddersfield, UK.

The areas surveyed were as follows: (1) **demographics** [11], including age, gender, ethnicity, current educational level, and relationship status. (2) **Patient health questionnaire (PHQ-9)** [17], a self-administered screening questionnaire for depression with nine questions cover different aspects of depression on a four-point scale from “0” (not at all), to “3” (nearly every day). The total score, used as the dependent variable in this research, can be categorised as 0–4 none, 5–9 mild, 10–14 moderate, 15–19 moderately severe, 20–27 severe. (3) **Generalised anxiety disorder questionnaire (GAD-7)** [18], a self-administered screening questionnaire for anxiety, with seven questions rated on the same four-point scale as the PHQ-9. Total score, also used as the dependent variable, takes 5, 10, and 15 as the cut-off points for mild, moderate and severe anxiety, respectively. (4) **Brief resilience scale (BRS)** [19], measures the ability to bounce back from stress, with a 5-point Likert response scale, for six items, ranging from 1 = strongly disagree to 5 = strongly agree. Unlike previous total scores, an averaged score is used instead, with 1.00 to 2.99 suggesting low resilience, 3.00 to 4.30 normal resilience and 4.31 to 5.00 high resilience. (5) **Brief mental wellbeing history** [11], asks about the students’ history of treatment and support for a mental health issue, including therapy and medication. (6) **EQ-5D-5 L** [32], a self-assessed, health related, quality of life measure with a 5-point Likert response scale. The overall score is used as a dependent variable, with the best health state coded as (11111) for a score of 5 and the worst health state (55555) being a score of 25. Additionally, the EQ-VAS [32] was also used for students to provide a broad self-assessment of their health, on a visual analogue scale ranging between 100 (best imaginable health) and 0 (worst imaginable health). (7) **COVID-19-related questions** [11]. A set of five COVID-19 questions were asked including: how often the person practised the recommended social distancing on a 5-point scale; the severity of the risk group the subject assumes they belong to; whether the subject is cohabiting with anyone falling with the risk groups; how likely the subject feels at the risk of contracting COVID-19; the extent to which the subject had felt needing support during lockdown (where ‘0’ was no need for extra support and ‘100’ indicated immediate support required).

Overall, this research adopts the exact data as initially explored, comprising 1173 subjects, 17 independent predictors and 6 decision variables [11]. The distributions of independent variables can be found in Table 1 with the descriptive analysis on six decision variables in Table 2, and detailed distributions of PHQ9, GAD7 and BRS6 in Table 3. The recent analysis [11] followed that as dominantly done in analysing questionnaire data using bivariate associations analysis and regression analysis. That is, for each individual predictor, the bivariate associations analysis adopts the statistical T-test to identify if the dichotomised answers are statistically significant towards a certain mental wellbeing state. The predictors that are showing statistical significance towards a given dependent variable then serve as input to the regression model, which analyses the contributions of these predictors through learned model coefficients. It is also worth noting that the independent use of bivariate associations analysis to exclude insignificant predictors might not work well in scenarios whereby a certain variable may not show statistical significance on its own, but may still contribute in combination with other variables in later regression analysis. Also as previously discussed, the dominant use of regression analysis may be constrained by its potential drawbacks such as sensitivity to outliers and situations where multiple highly correlated variables, as well as the reduced generalisation capability in the popular choice of linear regression that is limited in working with non-linear scenarios. These observations again motivate the underlying work to investigate the effectiveness of machine learning algorithms through a feature permutation importance strategy in analysing this questionnaire survey.

**Table 1:** Distributions of independent predictors [11]

Question/variable	Choice	*N * (%)
I. Demographics		
1, Gender	Female	826 (70.4%)
	Male	340 (29.0%)
	Others	7 (0.6%)
2, Ethnic origin	White	788 (67.2%)
	Non-white	385 (32.8%)
3, Education level	Undergrads	551(47.0%)
	Postgrads	622(53.0%)
4, Relationship	Single	529 (45.1%)
	Non-single	63 5(54.1%)
	Others	9 (0.8%)
II. Lifestyle / living situation		
1, During pandemic, have you suffered one or more from any of the following situations: worse personal relations, you or a loved one requiring hospitalisation, worse living conditions, loss of employment by you or your partner, worse financial situation, cancellation of an important event, death of a partner / close relative / friend	One or more adversities	979(86.6%)
2, How often have you been exercising during the lockdown period?	Very Often/Often	335(30.3%)
3, How often have you consumed alcohol during the lockdown period?	Always/Often	211(18.0%)
4, How often have you smoked tobacco during the lockdown period?	Always/Often	141(12.0%)
5, Has the current COVID-19 outbreak impacted your relationships with your friends/family?	Yes/Maybe	848 (72.3%)
6, How often have you been communicating with friends /family during the lockdown period?	Always/Often	709(60.4%)
III. Brief mental wellbeing history		
1, Have you ever been referred to, or participated in talking therapies for a mental health issue?	Yes	475 (40.5%)
2, Have you ever, or are you currently, taking medications for a mental health issue?	Yes, Previously/ Currently	325(27.7%)
3, Have you attempted to or accessed healthcare services for yourself or families during pandemic?	Yes	401(34.2%)
IV. COVID-19-related questions		
1, During pandemic how often have you practised recommended social distancing guidelines?	Always/Often	1096 (93.4%)
2, Which of these 3 COVID-19 risk groups do you do you believe you are under?	High/increased risk	175(14.9%)
3, Are you cohabiting with another person that falls within any of these risk groups?	Yes	353(30.1%)
4, How likely do you feel you are at risk of contracting COVID-19	Likely/extremely likely	194(16.5%)

**Table 2:** Descriptive analysis on decision variables (*N* = 1173) [11]

Variable	Mean ± STD
PHQ9 depression	10.91 ± 6.18
GAD7 anxiety	8.87 ± 5.80
BRS6 resilience	3.06 ± 0.31
EQ5D5L quality of life	7.91 ± 2.76
Health score	69.51 ± 20.62
Support needs	29.92 ± 30.08

**Table 3:** Detailed distributions of PHQ9, GAD7 and BRS6 [11]

PHQ9	[0-4] (none)		[5-9] (mild)		[10-14] (moderate)		[15-19] (moderately severe)		[20-27] (severe)	
	#	%	#	%	#	%	#	%	#	%
Male	85	25.0	101	29.7	72	21.2	54	13.1	28	8.2
Female	99	12.0	258	31.2	220	26.6	166	20.1	83	10.0
Whole	184	15.8	359	30.8	292	25.0	220	18.9	111	9.5

GAD7	[0-5] (none)		[6-10] (mild)		[11-15] (moderate)		[16-21] (severe)			
	#	%	#	%	#	%	#	%		
Male	144	42.4	97	28.5%	51	15.0	48	14.1		
Female	236	28.6	272	32.9%	157	19.0	161	19.5		
Whole	380	32.6	369	31.6%	208	17.8	209	17.9		

BRS6	[1, 2.99] (low resilience)		[3.00, 4.30] (normal resilience)		[4.31, 5.00] (high resilience)					
	#	%	#	%	#	%				
Male	92	27.1	248	72.9	0	0				
Female	215	26.0	611	74.0	0	0				
Whole	307	26.3	859	73.7	0	0				

## Methods

This work aims to investigate the significance of predictors towards each of the six dependent variables of interest, i.e. PHQ9 for depression, GAD7 for anxiety, BRS6 for resilience, EQ5D5L for quality of life, as well as a self-assessed health score and support needs. In order to illustrate the use of feature permutation importance to quantitatively assess the impact of independent predictors, this section takes PHQ9 as one of the six decision variables as an example to introduce the proposed methodology. The data *X* consists of a set of independent variables, which remains the same regardless of the choice of a particular decision variable, where $X_i (i=1, 2,..., 17)$ denotes *i*th independent variable; and $y_\text {PHQ9}$ is the dependent variable for the example, which is calculated as the sum of nine individual scores of the PHQ9 measure. $X^j$ and $y^j_{PHQ9}$ refer to the specific values entered by the *j*th $(j=1, 2,..., 1173)$ subject.

Recent literature typically employs all available data to train a computational model (regression in many cases), followed by analysing feature significance on this trained model, which could potentially lead to biased results [33]. Instead this research adopts the k-fold cross-validation (k-CV) [34] where different portions of the data are used to train and test a model on different iterations. In particular, the tenfold CV [34] is adopted whereby the full data, after a random shuffle, are evenly chopped into 10 subsets; in each iteration, one subset remains as the test data to compute feature significance, the remaining data are used to train a machine learning model. The overall result for a full 10-CV will be all 10 individual test results combined. To reduce variability and get less biased results, this work further repeats the 10-CV process 10 times, by randomly shuffling the whole data repeatedly each time that would lead to a potentially different partition [33]. The feature significance with respect to a certain machine learning model are finally averaged over the 10*10 iterations [34].
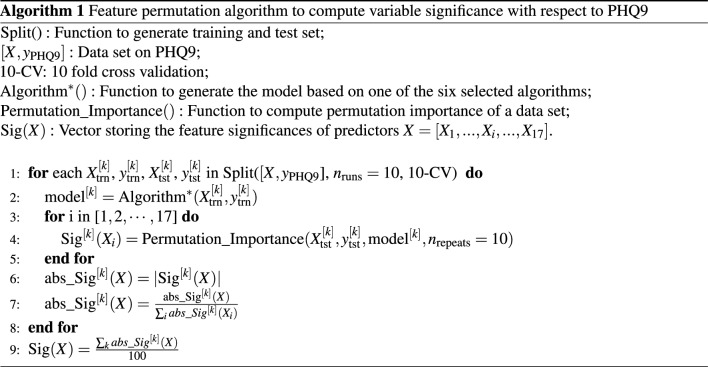


Algorithm 1 demonstrates the computational framework with details justified as follows. In the for-loop that executes commands an given number of times, each pair of the training data ($X^{[k]}_\text {trn}$ and $y^{[k]}_\text {trn}, k=1, 2,... 100$) and test data ($X^{[k]}_\text {tst}$, $y^{[k]}_\text {tst}$) are sampled out of the 10 repeats of 10-CV through the Split() function. The $X^{[k]}_\text {trn}$ and $y^{[k]}_\text {trn}$ are then used to train a machine learning model at certain iteration *k*. It is worth clarifying that all 6 dependent variables are of a continuous scale; hence a machine learning model fits the training data by minimising the mean squared errors (MSE) between the observed targets in the dataset, and the predictions computed by the underlying model. As one of the fundamental motivations for this research to investigate feature significance under a range of machine learning models that come with different learning strategies. The following algorithms, which have been briefly reviewed in Introduction are selected, i.e. the Linear Regression, the K Nearest Neighbours (KNN), the Support Vector Machine (SVM), the Decision Tree, the Random Forest, the Gradient Boosted Machine.

Once the $\text {model}^{[k]}$ is fit, the permutation importance of a feature can be calculated as: 1) compute a baseline metric of MSE evaluated on the test set ($X^{[k]}_\text {tst}$, $y^{[k]}_\text {tst}$), instead of the training set, which is a standard practice in machine learning to evaluate the generalisation capability of a model using a held-out set; 2) shuffle the values of a certain feature also on the test set, and evaluate the model performance using the shuffled data; 3) calculate the performance difference between the baseline metric and metric from permutating this feature column—this forms the permutation importance of the given feature. The permutation_importance() function summarises this procedure, where $n_\text {repeats = 10}$ suggests the each feature is randomly permutated 10 times to further reduce variability. Then return the feature to original values, and repeat this process with the next feature until all remaining independent variables; and finally returns vector $Sig^{[k]}(X)$ that represents the significance of the variable set in the *k*-th run.

It is worth noting that the significance of each feature calculated by permutation_importance() function can be a negative value that suggests the model performance decreases when the feature values increases. However, there could exist cases where one feature exhibits positive values sometimes but negative in other occasions possibly as a result of the change of particular data split used for a certain run. Hence, to minimise this impact for computing a more objective overall feature importance across multiple random runs, all feature significances as a result of permutation_importance() are forced to take their absolute values to avoid the cancellation of positive and negative values, resulting in the $\text {abs}\_\text {Sig}^{[k]}(X)$ vector. In order to ease averaging results across multiple runs later, this is followed by a normalisation step within each single run, such that the sum of the feature significance of each feature is added to $\sum _i |abs\_Sig^{[k]}(X_i)| = 1$. Finally, the overall feature significance is averaged over all iterations.

## Discussion

**Table 4:** Feature significance with respect to PHQ9 using the permutation importance approach

*PHQ9*	Regression	KNN	SVM	Decision tree	Random forest	Gradient boost	Average
Gender	0.040	0.063	0.045	0.058	0.054	0.054	0.052
Ethnics	0.036	0.061	0.025	0.056	0.044	0.031	0.042
Education	0.039	**0.069**	0.026	0.059	0.052	0.039	0.047
Relationship	0.032	**0.065**	0.040	0.058	0.047	0.035	0.046
Adversity	**0.080**	0.056	0.059	0.054	**0.067**	**0.068**	**0.064**
Exercise	**0.148**	**0.080**	**0.165**	**0.085**	**0.111**	**0.133**	**0.120**
Alcohol	0.009	0.044	0.019	0.035	0.029	0.023	0.026
Tobacco	0.053	0.041	0.022	0.046	0.038	0.047	0.041
Relation-Impact	**0.191**	**0.079**	**0.210**	**0.096**	**0.133**	**0.176**	**0.148**
Communication	**0.113**	**0.085**	**0.097**	**0.077**	**0.092**	**0.123**	**0.098**
Therapy	**0.095**	0.062	**0.092**	**0.065**	0.055	**0.067**	**0.073**
Medication	0.053	0.055	**0.064**	**0.069**	0.056	0.049	0.058
Health-service	0.046	0.065	0.053	0.062	**0.059**	0.044	0.055
Social-distancing	0.017	0.028	0.013	0.031	0.028	0.042	0.026
Risk-group	0.010	0.041	0.015	0.045	0.040	0.017	0.028
Living-group	0.029	0.063	0.038	0.064	0.058	0.033	0.047
Contract-risk	0.008	0.044	0.017	0.042	0.037	0.020	0.028

The top-5 most significant features highlighted in bold

This section discusses results generated through the proposed feature permutation importance. Tables 4, 5, 6, 7, 8 and 9 summarise the results for each of the six decision variables of interest, whereby each column refers to one of the six selected machine learning algorithms; each row specifies a predictor; each entry $Sig(X_i^{[j]})$, which is calculated as a result of averaging over 10 random runs out of 10-CV by Algorithm 1, denotes the importance of predictor *i* under machine learning model *j*. The implementation of these machine learning algorithms with all default choices of hyperparameters is provided by Sklearn package [34], which is a free machine learning library for the Python programming language. It can be verified that the sum of importance of each predictor for algorithm *j* is added up to the unit value $\sum _iSig(X_i^{[j]})=1$.

**Table 5:** Feature significance with respect to GAD7 using the permutation importance approach

*GAD7*	Regression	KNN	SVM	Decision tree	Random forest	Gradient boost	Average
Gender	0.051	0.064	0.044	0.059	0.053	0.051	0.054
Ethnics	0.034	**0.069**	0.023	0.053	0.051	0.023	0.042
Education	0.019	**0.071**	0.027	0.060	0.058	0.033	0.045
Relationship	0.011	0.065	0.023	0.058	0.053	0.029	0.040
Adversity	**0.080**	0.049	**0.077**	0.051	**0.068**	**0.094**	**0.070**
Exercise	**0.053**	0.060	0.059	0.059	**0.062**	0.056	0.058
Alcohol	0.008	0.048	0.016	0.042	0.035	0.027	0.029
Tobacco	**0.072**	0.038	0.053	0.043	0.038	0.054	0.050
Relation-Impact	**0.222**	**0.087**	**0.208**	**0.085**	**0.127**	**0.198**	**0.154**
Communication	**0.136**	**0.080**	**0.122**	**0.081**	**0.100**	**0.127**	**0.108**
Therapy	**0.146**	0.065	**0.126**	**0.092**	**0.072**	**0.090**	**0.099**
Medication	0.049	0.060	**0.063**	**0.072**	0.061	0.043	0.058
Health-service	0.065	**0.067**	0.057	**0.067**	0.061	**0.061**	**0.063**
Social-distancing	0.008	0.025	0.013	0.030	0.023	0.024	0.020
Risk-group	0.012	0.043	0.025	0.051	0.047	0.032	0.035
Living-group	0.020	0.062	0.031	0.052	0.053	0.025	0.041
Contract-risk	0.015	0.047	0.032	0.045	0.041	0.034	0.036

The top-5 most significant features highlighted in bold

Results on the use of regression model is first discussed, as it was also used in the initial research [11] over the exact same data, though it is worth noting again the proposed permutation importance also utilises regression as the base model, its calculation of predictor significance is being entirely model agnostic that does not rely at all coefficients generated by the regression model. Due to limited space, results as reported in [11] that serve a comparison basis are not directly presented here, but it is worth recalling again that [11] followed a common two-step approach in analysing questionnaire data, whereby the bivariate associations analysis is first adopted through the statistical T-test to identify if the dichotomised answers are statistically significant towards a certain mental wellbeing state; predictors that are showing statistical significance then serve as input to the regression analysis, which computes contributions of these predictors through learned model coefficients.

For regression over PHQ9, as summarised in Table 4, the top-5 most significant features highlighted in bold are impact of relationship, exercise frequency, communication frequency, history of talking therapy and adversity, four out of which are also shared by initial research [11]. Similar observations can be drawn for GAD7 in Table 5, where four out of five most important predictors are shared by both approaches, i.e. impact of relationship, history of talking therapy, communication frequency and adversity. It is worth recalling again that the proposed approach works on the full set of all 17 predictors; whereas research[11] only considers predictors that are of statistical significance through an independent bivariate association analysis on individual variable. This makes the two-step analysis disjoint and possess risks of removing predictors that are not statistically significant on its own, but might be so when considered with other predictors. Such drawback of conducting an independent bivariate association analysis first may be exemplified with the analysis of BRS6, where the initial research [11] removed 14 predictors with only 3 kept for regression analysis, whereas the proposed approach is still able to work on the full set of predictors.

**Table 6:** Feature significance with respect to BRS6 using the permutation importance approach

*BRS6*	Regression	KNN	SVM	Decision tree	Random forest	Gradient boost	Average
Gender	0.060	**0.067**	0.064	**0.069**	**0.090**	**0.080**	**0.072**
Ethnics	**0.184**	**0.074**	**0.079**	**0.089**	**0.078**	**0.116**	**0.103**
Education	0.055	**0.068**	0.060	0.055	0.061	0.052	0.058
Relationship	0.014	**0.077**	**0.080**	0.064	**0.085**	0.057	0.063
Adversity	**0.087**	0.053	0.065	0.066	**0.070**	**0.079**	**0.070**
Exercise	0.052	0.065	**0.077**	**0.071**	0.062	0.051	0.063
Alcohol	0.015	0.044	0.036	0.036	0.038	0.034	0.034
Tobacco	0.017	0.039	0.041	0.037	0.032	0.043	0.035
Relation-Impact	0.015	0.066	**0.070**	0.058	0.059	0.043	0.052
Communication	**0.108**	**0.070**	**0.078**	**0.079**	**0.082**	**0.096**	**0.086**
Therapy	**0.103**	0.061	0.058	0.055	0.054	0.045	0.063
Medication	0.026	0.055	0.057	0.055	0.044	0.046	0.047
Health-service	**0.124**	0.065	0.057	**0.069**	0.070	**0.078**	**0.077**
Social-distancing	0.023	0.029	0.031	0.025	0.023	0.030	0.027
Risk-group	0.042	0.053	0.045	0.063	0.050	0.044	0.050
Living-group	0.018	0.064	0.058	0.064	0.056	0.068	0.055
Contract-risk	0.057	0.051	0.044	0.045	0.046	0.039	0.047

The top-5 most significant features highlighted in bold

As for EQ5D5L, there are also four out of top-5 significant predictors shared by both approaches, being history of health services attempt, medication and talking therapy as well as risk group. For quality of life, apart from the risk group, all three items in the wellbeing history category of the questionnaire selected as most important predictors for both approaches. In terms of self-assessed health score, three out of five top significant predictors are shared by both approaches, i.e. history of health services attempt and talking therapy, and frequency of communication with families/friends. For support needs, ethnics, history of medication and health services attempts are among the top-5 shared predictors. In a nutshell, for 5 decision variables (BRS6 is not applicable), 18 out of 25 top-5 predictors selected by the proposed approach are also shared by the two-step statistics and regression approach [11]—this suggests, despite of different mechanisms of computing factor importance, variables that are of intrinsic significance can be captured by data-driven methods of different kinds. Of course, the proposed work comes with the added capability of working with decision variable like BRS6 to look at the whole set of predictors from a holistic view when individual predictor does not come with statistical significance.

To analyse these results further, for PHQ9, exercise, relationship impact and communication with families/friends are all among the most predictors as highlighted in bold across all 6 different learning algorithms. In order to give a more succinct and overall assessment across the full set of learning algorithms, the importances of individual predictor are averaged to compute an overall importance of predictor as shown in the last column across Tables 4, 5, 6, 7, 8 and 9. As the sum of feature importances under each column/algorithm is the unit value, the sum of each averaged feature significance is also guaranteed to 1 in the ’Average’ column, thus easing the interpretation. For PHQ9, with relationship impact, exercise and the level of communication uniformly selected by all algorithms, it is not a surprise to see them remain the top 3, followed by history of talking therapy and experience of adversities.

For GAD7, as shown in Table 5, the relationship impact and the level of communication have again been uniformly selected by all 6 learning algorithms. Different from PHQ9, where exercise contributes more towards regulating depression level, the role of exercise for anxiety is not as important, but still remains the 6th most important factor. This suggests that five out of top-6 most important factors are shared by both PHQ9 and GAD7, i.e. adversity, relationship impact, communication level and talking therapy history and exercise. These observations suggest exercising frequently while maintaining regular communications with families and friends to keep good relationship could contributes significantly towards low level of depression and anxiety. Different from PHQ9 and GAD7, where the top-five predictors combined contribute around 50% significance, significance of predictors for BRS6 is more sparsely and relatively evenly distributed, with their top-5 predictors combined only contributing around 40% importance, suggesting that a more diverse set of factors could impact the resilience level. While the communication frequency still remains one consistently significant factor across dependent variables analysed so far, it is interesting to identify ethnics origin also contributes significantly across all algorithms, especially the particularly high coefficient from the regression model.

**Table 7:** Feature significance with respect to EQ5D5L using the permutation importance approach

*EQ5D5L*	Regression	KNN	SVM	Decision tree	Random forest	Gradient boost	Average
Gender	0.016	0.054	0.030	0.054	0.048	0.046	0.042
Ethnics	0.008	0.060	0.025	0.060	0.057	0.026	0.039
Education	0.013	0.062	0.027	0.051	0.046	0.025	0.037
Relationship	0.012	0.064	0.028	0.047	0.046	0.032	0.038
Adversity	0.045	0.049	0.075	0.068	0.047	0.048	0.055
Exercise	0.096	0.062	**0.091**	0.060	0.062	**0.099**	0.078
Alcohol	0.021	0.038	0.023	0.029	0.030	0.037	0.030
Tobacco	0.032	0.042	0.031	0.029	0.037	0.024	0.033
Relation-Impact	**0.133**	**0.075**	**0.153**	**0.070**	**0.090**	**0.132**	**0.109**
Communication	0.069	**0.070**	0.063	0.057	0.063	0.053	0.063
Therapy	**0.180**	**0.091**	**0.150**	**0.106**	**0.115**	**0.144**	**0.131**
Medication	**0.117**	**0.067**	**0.082**	**0.091**	**0.077**	0.070	**0.084**
Health-service	**0.108**	**0.076**	**0.090**	**0.079**	**0.092**	**0.092**	**0.090**
Social-distancing	0.008	0.026	0.012	0.030	0.023	0.033	0.022
Risk-group	**0.123**	0.061	0.070	0.066	**0.084**	**0.091**	**0.083**
Living-group	0.016	0.055	0.030	**0.068**	0.053	0.033	0.043
Contract-risk	0.004	0.046	0.019	0.032	0.029	0.015	0.024

The top-5 most significant features highlighted in bold

**Fig. 1:**
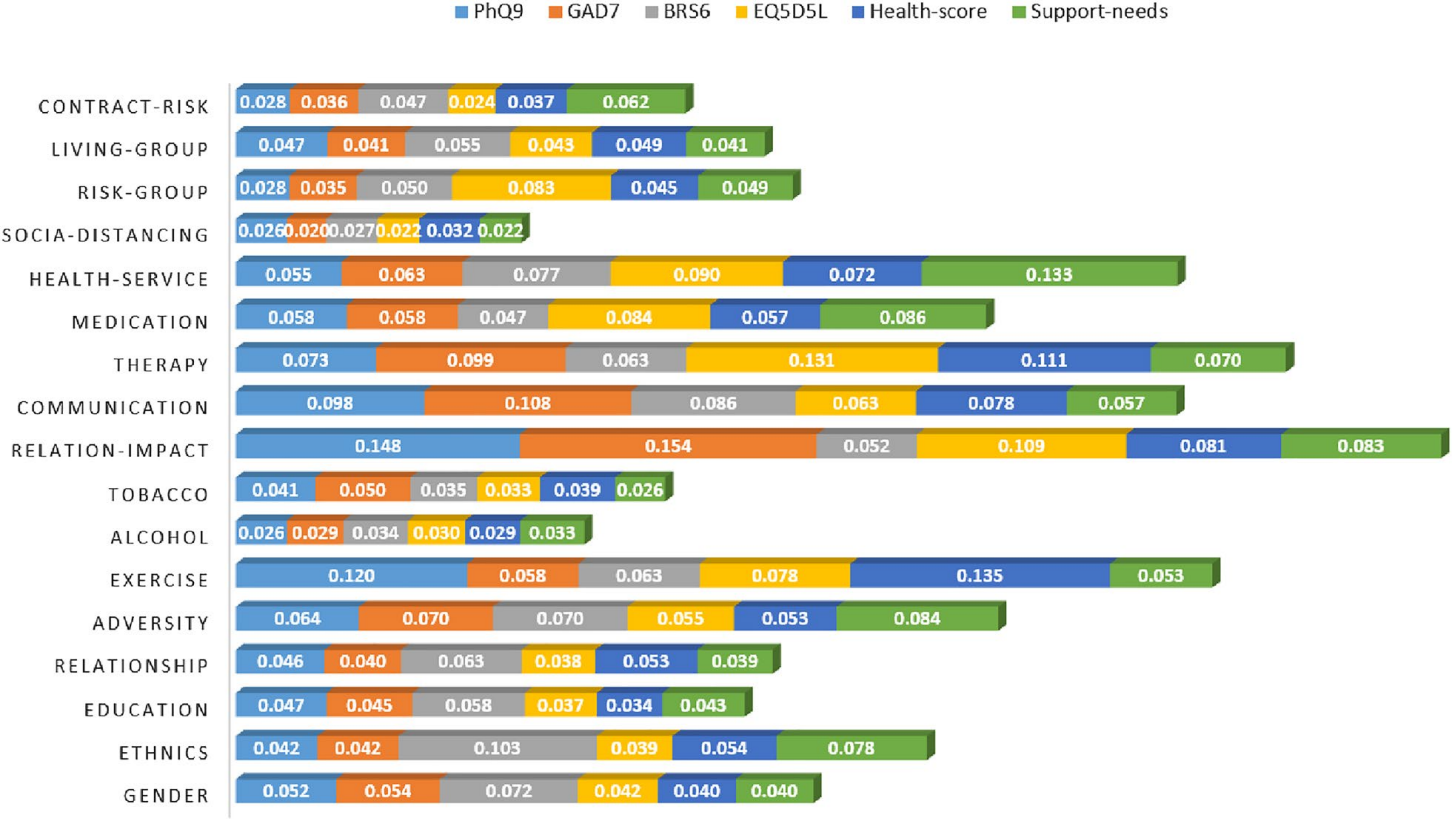
Visualisation of overall feature significance

For EQ5D5L on life quality, the full set of medical history, including talking therapy, medication and access to health services have all been selected among top factors across all algorithms. This observation suggests the personal medical history and wellbeing have a direct impact on quality of life, though it could also be largely influenced by relationship with families and friends, a factor that has been instrumental for both the anxiety and depression state. As for the self-administered health score, the relationship impact, communication frequency and history of talking therapy, remain the most important factors, which are also shared by both PHQ9 and GAD7. The exercise and access to health services are also shared as one of the top factors by PHQ9 and GAD7, respectively. These observations amplifies the significance of the set of shared factors in studying multiple mental wellbeing states. In terms of extra support needs under the pandemic, apart from the experience of adversity, the significant factors also come from personal medical wellbeing including the history of medicine and access to healthcare services, as well as the impact of relationship with families and friends.

Overall, the importance of each factor towards each of the decision variable can be summarised in Fig. 1, where each horizontal bar summarises the contributions of the underlying predictor in the corresponding decision variable as previously calculated in Tables 4, 5, 6, 7, 8 and 9. Vertically, as an example, it is clear to visualise in Fig. 1 that Relation-impact has the biggest bar in blue, indicating the most significant factor for PHQ9. Horizontally, the total length of each horizontal bar represents the accumulated significance of each factor across all decision variables. This empowers to obtain an holistic and unified view of the most significant factors across numerous computing mechanisms and variables of interest. In the context of student mental wellbeing, where multiple elements such depression, anxiety and resilience may be concerned, while considering different factors may play different roles in related decision variables, the proposed approach provides a framework that can highlight the most important factors after considering numerous computing algorithms. In our case study, the relationship impact and communication with families/friends, the history of talking therapy and access to healthcare services, as well as the exercise remain the overall top-5 factors across the six decision variables for mental wellbeing of university students. Of course, such results may require further examination by decision-makers, but they do provide brief key information for stakeholders to reflect promptly especially under unprecedented situations like COVID19; e.g., the higher education provider might consider plans and actions to improve student relationship and communication with family and friends, which are the most important factors to improve university student mental wellbeing from multiple perspectives as identified in this paper.

## Conclusion

This paper has proposed the use of multiple advanced machine learning algorithms under the permutation importance strategy to analyse the questionnaire online data in the context of mental wellbeing of university students. In comparison with the dominantly applied regression for questionnaire analysis, which is also often supported with a prior an independent bivariate association analysis to identify predictors of statistical significance, both the permutation approach and regression method identify a large set of shared predictors in the study of multiple mental wellbeing states—while there may not exist an absolutely objective approach in identifying universally agreed factors, simply due to that certain factors may be interpreted differently by individuals and that different computing methods quantify the importance of a variable differently owing to the use of different learning theory; the fact that many of the identified predictors are shared by both approaches, still highlights the intrinsic importance of these factors. Furthermore, another advantage of using the permutation approach empowers access to a diverse set of algorithms originating from different learning frameworks, including those without being able to provide inherent coefficients to suggest quantitative contributions. The outcomes from different computational algorithms that provide potentially different views could be aggregated further across a diverse set of algorithms to identify most contributing factors from a more holistic perspective, potentially making the generated results more reliable.

In our investigation of student mental wellbeing in a UK institution, the subset of relationship impact, communication frequency, history of talking therapy, exercise frequency, access to health services, experience of adversity have been found as common significant factors contributing to depression, anxiety and self-administered health score. Whereas a more diverse set of factors could potentially influence personal resilience, the life quality instead is significantly impacted by personal medical history and wellbeing state, which also influence the support needs. While the historical medical record and wellbeing history may differ with individuals and the occurrence of personal adversity is more unpredictable, frequent communication with families and friends to keep good relationship as well as regular exercise are generally contributing to better personal wellbeing. Whilst promising, further investigation of more advanced machine learning algorithms in computing and interpreting feature significance as well as the exploration of feature importance in improving the accuracy of supervised learning remain interesting future work.

**Table 8:** Feature significance with respect to Health-score using the permutation importance approach

*Health-score*	Regression	KNN	SVM	Decision tree	Random forest	Gradient boost	Average
Gender	0.026	0.059	0.030	0.043	0.046	0.039	0.040
Ethnics	0.051	0.062	0.040	0.063	0.058	0.049	0.054
Education	0.005	0.062	0.015	0.057	0.046	0.019	0.034
Relationship	0.037	0.063	0.033	0.062	0.056	**0.066**	0.053
Adversity	0.068	0.042	0.070	0.043	0.040	0.056	0.053
Exercise	**0.170**	**0.094**	**0.159**	**0.090**	**0.130**	**0.167**	**0.135**
Alcohol	0.006	0.046	0.021	0.043	0.036	0.020	0.029
Tobacco	0.046	0.042	0.030	0.043	0.034	0.040	0.039
Relation-Impact	**0.081**	**0.064**	**0.135**	0.065	**0.066**	**0.073**	**0.081**
Communication	**0.092**	**0.075**	**0.095**	**0.069**	**0.071**	**0.069**	**0.078**
Therapy	**0.153**	**0.074**	**0.114**	**0.095**	**0.095**	**0.132**	**0.111**
Medication	0.043	0.063	0.076	0.060	0.054	0.048	0.057
Health-service	**0.077**	**0.066**	**0.095**	**0.068**	**0.067**	0.059	**0.072**
Social-distancing	0.028	0.031	0.013	0.042	0.032	0.044	0.032
Risk-group	0.049	0.050	0.014	0.048	0.061	0.050	0.045
Living-group	0.025	0.060	0.042	**0.068**	0.065	0.033	0.049
Contract-risk	0.042	0.047	0.016	0.041	0.041	0.036	0.037

The top-5 most significant features highlighted in bold

**Table 9:** Feature significance with respect to Support-needs using the permutation importance approach

*Support-needs*	Regression	KNN	SVM	Decision tree	Random forest	Gradient boost	Average
Gender	0.019	0.059	0.024	0.053	0.051	0.037	0.040
Ethnics	**0.102**	**0.066**	0.082	**0.064**	0.063	**0.094**	**0.078**
Education	0.016	**0.069**	0.013	0.063	**0.066**	0.031	0.043
Relationship	0.009	0.063	0.015	0.060	0.063	0.024	0.039
Adversity	**0.109**	0.045	**0.097**	0.064	**0.069**	**0.120**	**0.084**
Exercise	0.044	0.051	0.058	0.063	0.060	0.042	0.053
Alcohol	0.016	0.044	0.013	0.048	0.044	0.034	0.033
Tobacco	0.014	0.040	0.015	0.037	0.031	0.019	0.026
Relation-Impact	**0.090**	0.064	**0.128**	**0.070**	**0.068**	**0.079**	**0.083**
Communication	0.051	0.063	0.052	0.064	0.066	0.046	0.057
Therapy	0.051	**0.076**	**0.113**	**0.064**	0.058	0.059	0.070
Medication	**0.113**	**0.073**	**0.098**	**0.075**	**0.071**	**0.088**	**0.086**
Health-service	**0.186**	**0.088**	**0.183**	**0.090**	**0.102**	**0.149**	**0.133**
Social-distancing	0.016	0.030	0.011	0.026	0.021	0.025	0.022
Risk-group	0.058	0.047	0.023	0.055	0.047	0.064	0.049
Living-group	0.021	0.064	0.027	0.052	0.058	0.023	0.041
Contract-risk	0.084	0.056	0.048	0.052	0.062	0.068	0.062

The top-5 most significant features highlighted in bold

## Data Availability

The datasets generated and/analysed during the current study are not publicly available, because of confidential nature of university student information. Data are however available from the corresponding author on reasonable request and with permission of the University of Huddersfield, School of Computing and Engineering Institutional Data Access/Ethics Committee.
